# Nuclear and Cytoplasmic Soluble Proteins Extraction from a Small Quantity of *Drosophila*’s Whole Larvae and Tissues

**DOI:** 10.3390/ijms160612360

**Published:** 2015-06-01

**Authors:** Luca Lo Piccolo, Rosa Bonaccorso, Maria Cristina Onorati

**Affiliations:** Dipartimento di Scienze e Tecnologie Biologiche, Chimiche e Farmaceutiche (STEBICEF), Viale delle Scienze, Universita’ degli Studi di Palermo, Palermo 90128, Italy; E-Mails: lucalopiccolo@gmail.com (L.L.P.); rosabonaccorsog@gmail.com (R.B.)

**Keywords:** Drosophila, hnRNPs, proteins extraction

## Abstract

The identification and study of protein’s function in several model organisms is carried out using both nuclear and cytoplasmic extracts. For a long time, *Drosophila*’s embryos have represented the main source for protein extractions, although in the last year, the importance of collecting proteins extracts also from larval tissues has also been understood. Here we report a very simple protocol, improved by a previously developed method, to produce in a single extraction both highly stable nuclear and cytoplasmic protein extracts from a small quantity of whole *Drosophila*’s larvae or tissues, suitable for biochemical analyses like co-immunoprecipitation.

## 1. Introduction

In the past decades, *Drosophila* has represented the preferred model system for genetics and developmental biology studies. Our understanding about the cellular development mechanisms and the genetic pathways are principally due to works in *Drosophila*. Indeed, in the last years, the potential of *Drosophila* as an organism for biochemical studies, in particular to study structure and function of proteins, has also been revealed. To date, for large-scale nuclear and cytoplasmic extract preparations, all the protocols use *Drosophila*’s embryos as a source of material [1,2], because collection of *Drosophila*’s embryos is relatively easy. Going from a single embryo up to several mg of embryos, it is also possible to obtain a large amount of proteins involved in a variety of cellular processes and activities [3,4]. On the other hand, nuclear and cytoplasmic protein extracts are an excellent source of material suitable for a wide variety of applications, such as mRNA splicing, gene expression studies, RNA binding or *in vitro* transcription [1,2,5]. The knowledge of the *Drosophila*’s proteins function came out from studies conducted in different *Drosophila*’s tissues like mitotic chromosomes in larval brains, polytene chromosomes staining in salivary glands as well as cell cycle profiles in different tissues, but only few protocols utilize *Drosophila*’s larvae or larval tissues as good source of proteins [6].

For that reason, we decided to improve a protocol previously developed to produce highly stable large-scale protein nuclear extracts from whole *Drosophila*’s larvae [7]. We performed a new method, which allows the production of nuclear protein extracts, starting from a small number of *Drosophila*’s Whole Larvae (WL) or tissues like Salivary Glands (SG), Brains (B), and Malpighian Tubuli (MT). Interestingly, in the same extraction we are also able to produce cytoplasmic extracts avoiding any contamination from nuclear proteins. The yield of these extracts is considerable for both nuclear and cytoplasmic proteins. Finally, this protocol also offers the great advantage to monitor those proteins that move between nucleus and cytoplasm such as Argonaute 2 (AGO2), member of the Argonaute/PIWI (P-element induced wimpy testis) protein family [8] or heterogeneous nuclear Ribonucleoproteins (hnRNPs) that are known to shuttle between nuclear and cytoplasmic compartments [9,10].

## 2. Results

**Figure 1:**
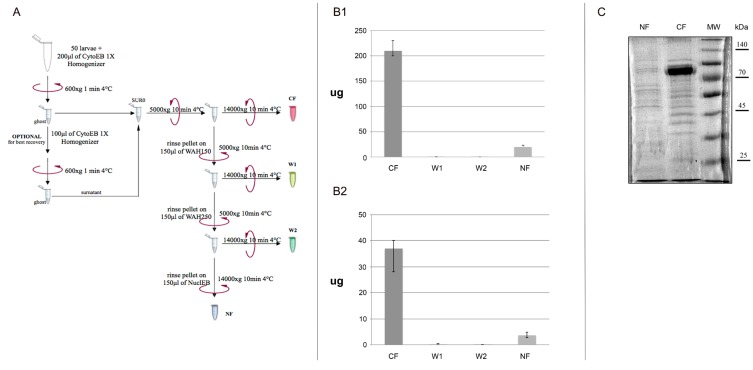
Workflow’s chart. (**A**) Graphic representation of our protocol; All four fractions extracted are assayed in a Bradford reaction to estimate the total yield of proteins extracted for each extraction in Whole Larvae (WL) (**B1**) and in tissues (**B2**). CF: Cytoplasmic fraction; W1: supernatant obtained after washing with WASH150 solution; W2: supernatant obtained after washing with WASH250 solution; NF: Nuclear fraction. Standard error is obtained from independent mensuration of six different extractions; (**C**) 3 μg of NF and CF samples extracted from WL are loaded in 10% acrylamide gel to monitor fractioning of the protein extraction procedures.

Solution and glassware are pre-chilled on ice before use and in order to minimize protein degradation, all steps in this protocol have been carried on ice. About 20 to 30 third instar WL (or 60–70 couple of SG, 60 B or 60 MT tissues), grown on standard corn medium at 25 °C, are transferred on petri capsule, washed with NaCl 0.7% and then transferred on 1.5 mL Eppendorf tube. Larvae are bulk-homogenized with a sterile Polypropylene (PP) pestle in 200 μL of Cytoplasmic Extraction Buffer 1X (CytoEB1X). The ice-cold homogenate is then centrifuged to spin down ghost larvae and the supernatant is carefully removed and processed in a further steps (Sur0) (Figure 1A). Optionally about 100 μL of CytoEB1X could be added on slurry ghost larvae and re-homogenized for a better proteins yield. About 150–200 μL of supernatant is centrifuged and the obtained supernatant is carefully recovered and clarified with a second centrifugation step obtaining thus Cytoplasmic Fraction (CF). The dried pellet is used for nuclear proteins extraction in further purification steps. To ensure that cells are completely lysed, two washing steps are performed with increased sucrose concentration using the two wash buffers WASH150 and WASH250. One hundred and fifty microliters of WASH150 are used to re-suspend pellet up and down avoiding bubbles formation. This solution is centrifuged and the obtained supernatant is carefully recovered and clarified with a second centrifugation step, keeping aside Wash1 (W1).

One hundred and fifty microliters of WASH250 were then used to re-suspend pellet up and down avoiding the bubbles formation. This solution is centrifuged and the obtained supernatant is carefully recovered and clarified with a second centrifugation step, keeping aside Wash2 (W2). Finally, 150 μL of Nuclear Extraction Buffer (NEB) are used to re-suspend the pellet. The high sucrose concentration and detergent in NEB buffer ensure the lysis of nuclear membrane. Vigorous vortexing is performed to ensure a complete solubilisation of the pellet. A centrifugation step is performed to recover the soluble protein fraction from lysed nuclei obtaining Nuclear Fraction (NF). We recapitulated all the steps of the method in a simple Workflow’s chart (Figure 1A).

The four soluble fractions CF, W1, W2 and NF are transferred in a 1.5 mL Protein LoBind Tube Eppendorf and stored at −20 °C. Bradford assay is used to quantify the protein concentration in each one of the four fractions for WL (Figure 1B1) as well as tissues (Figure 1B2). Moreover, 3 μg of NF and CF samples extracted from WL are loaded in 10% acrylamide gel to monitor fractioning of the protein extraction procedures (Figure 1C).

**Figure 2:**
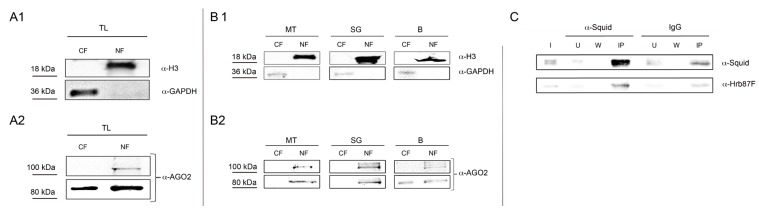
*In vitro* and *in vivo* analysis of proteins extracted from *Drosophila*’s small quantity of whole larva and tissues. Western Blot analysis of proteins extracted from WL to detect any contamination of cytoplasmic proteins in nuclear fractions and *vice versa*. The same amount (3 μg) of different protein fractions is used in an immunodetection assay with two different antibodies: anti-αH3 (1:4000) and anti-GAPDH (1:4000) (**A1**). The same extracts are used in an immunodetection assay with anti-AGO2 (1:2000) (**A2**); (**B1**) Western Blot analysis of proteins extracted from different *Drosophila*’s tissues like MT, SG and B. (**B2**) 3 μg of nuclear and cytoplasmic protein fractions are used in an immunodetection assay with anti-AGO2 (1:2000), anti-αH3 (1:4000), and anti-GAPDH (1:4000); (**C**) Co-immunoprecipitation of Squid and Hrb87F hnRNPs from nuclear extracts. The same amount of nuclear protein fractions is used in an immunoprecipitation assay with anti-Squid antibody and IgG. For the immunodetection assay anti-Squid (1:200) and anti-Hrb87F (1:4000) were used. I = Input, U^Squid^ = Unbound from anti-Squid IP, W^Squid^, wash from anti-Squid IP, U^IgG^ = Unbound from IgG IP, W^IgG^, wash from anti-IgG IP, IP^Squid^ = Immunoprecipitated material from anti-Squid, IP IP^IgG^ = Immunoprecipitated material from IgG IP.

In order to detect any contamination of cytoplasmic proteins in nuclear fractions and *vice versa*, we checked the different fractions by Western Blot analysis loading 3 μg of protein extracts for each fraction. As shown in Figure 2A1, we obtained cytoplasmic fraction free of nuclear proteins and *vice versa.* We used two different antibodies, the anti-H3 to monitor proteins in the nuclear fraction and the anti-GAPDH to monitor proteins in the cytoplasmic fraction. In order to test the efficacy of our protocol, we performed a western blot analysis on the same proteins extracts using an antibody against AGO2 protein, a member of the Argonaute/PIWI protein family. As shown in Cernilogar *et al.* [11], AGO2 is present in both the nucleus and the cytoplasm. In extracts from WL the 100 kDa isoform is only present in the nuclear fraction, while the 80 kDa isoform is present in both cytoplasmic and nuclear fraction (Figure 2A2). Thus our protocol is optimal to monitor those proteins that move between nuclear and cytoplasmic compartment.

In order to demonstrate that our protocol is suitable also for protein extracts from *Drosophila* tissues, we repeated the same analysis with proteins extracted from *Drosophila*’s larval tissues MT, SG and B. In all the cases, checking the different fractions by Western Blot analysis, loading 3 μg of protein extracts for each fraction, we obtained the same results for H3 and GAPDH proteins (Figure 2B1), with a good yield. Regarding AGO2 (Figure 2B2), the two isoforms of this protein are present in both fractions at least in B cells.

The nuclear and cytoplasmic extracts produced with our protocol are highly stable and are suitable not only for Western Blot, but also for co-immunoprecipitation analysis. We decided to test two heterogeneous nuclear Ribonucleoproteins (hnRNPs) Squid and Hrb87F, since, like several other RNA Binding Proteins (RBPs), these proteins are known to move between nucleus and cytoplasm, having important roles in both compartments [9,10]. Indeed, Squid and Hrb87F, are known to be engaged in the omega-speckles compartment. Omega speckles are specialized nuclear compartments, distinct from other nuclear speckles, localizing in the nucleoplasm close to chromatin edges and believed to function as storage sites for the unengaged hnRNPs and other related RNA-processing proteins [12,13]. As shown in Figure 2C, we co-immunoprecipitate from nuclear extract the two hnRNPs.

## 3. Experimental Section

### 3.1. Fly Strains

Flies were raised at 25 °C on K12 medium [14]. Oregon-R-P2 wild type fly strain (Bloo 2376) was obtained from Bloomington Drosophila Stock Center at Indiana University.

### 3.2. Protein Extraction

The chosen amount of third instar larvae or couple or number of tissues grown on standard corn medium at 25 °C, are transferred on petri capsule, washed with NaCl 0.7% and then transferred on 1.5 mL Eppendorf tube. Larvae are bulk-homogenized in 200 μL of CytoEB1X (CytoEB2X: 30 mM Hepes-KOH pH 7.6, 20 mM KCl, 10 mM MgCl_2_, 0.2 mM EDTA pH 8.0, 20% Glycerol).

To ensure that cells were completely lysed, two washing steps are performed with increased Sucrose concentration using 150 μL of WASH150 (CytEB 1X, 150 mM Sucrose) and 150 μL of WASH250 (CytEB 1X, 250 mM Sucrose). Finally, 150 μL for whole larvae extracts (or 50 μL for tissues extracts) of NEB **(**350 mM Sucrose, 15 mM Hepes-KOH pH 7.6, 385 mM KCl, 5 mM MgCl_2_, 0.1 mM EDTA pH 8.0, 0.05% Tween 20, 10% Glycerol) are used to re-suspend the pellet.

### 3.3. Antibodies

Mouse monoclonal antibodies and rabbit monoclonal antibodies against the following proteins are used at the indicated dilutions for WB: Hrb87F (P11 anti-mouse) [13] 1:100; Squid (1B11 Developmental Studies Hybridoma Bank anti-mouse) [15] 1:2000; anti-H3 (Upstate anti-rabbit) (1:4000), anti-GAPDH (Gene Tex anti-rabbit) (1:4000), anti-AGO2 [11] (1:2000).

### 3.4. Western Blot Analysis

Total proteins from WL, SG, MT and B tissues are extracted as described in this manuscript. The SDS-PAGE separated proteins are transferred onto nitrocellulose membrane (Whatman Schleicher and Schuell, Florham Park, NJ, USA) for Western detection using SuperSignal West Femto substrate (Pierce, Waltham, MA, USA). Chemiluminescent signals are acquired with the ChemiDoc XRS imager (BioRad, Hercules, CA, USA).

### 3.5. Co-Immunoprecipitation

For the co-immunoprecipitation assay, nuclear proteins extracts are prepared as described in this manuscript and 100 μg of nuclear protein extracts are used for each IP. Squid immunoprecipitation is performed on the nuclear protein extracts using 5 μL of Dynabeads protein A (Novex by life technologies AS, Oslo, Norway) and 4 μg of affinity purified Squid antibody (1B11). 4 μg of IgG anti-mouse (Santa Cruz Biotechnology, Dallas, TX, USA) is used as control. 5 μL of Dynabeads are re-suspended in 200 μL of Incubation Buffer (IB) (10 mM Hepes-KOH pH 8.0, 1 mM EDTA, 10% Glycerol, 50 mM NaCl) and washed three times for 10 min in gentle agitation. After the last wash, the beads are incubated in 75 μL of IMmuno Precipitation Buffer for Incubation (IMPBI) (10 mM Hepes-KOH pH 8.0, 10% Glycerol, 100 μg/mL PMSF, complete 1X, Roche diagnostics GmbH, Mannheim, Germany) with Squid antibody and IgG for 2 h at 4 °C in gentle agitation. The beads are washed three times in 200 μL of IB Buffer. Finally the beads are incubated with nuclear protein extract and with IMPBI Buffer (volume 1:1) over night in gentle agitation. The next day, unbound material is collected; the beads are washed three times for 10 min in IMmunoPrecipitation Buffer for wash (IMPBW) (10 mM Hepes-KOH pH 8, 100 mM NaCl, 0.05 tween 20, 10% Glycerol, 100 μg/mL PMSF, complete 1X, Roche). The first wash is collected. Finally, the bound material is eluted using 30 μL SDS-PAGE sample buffer and all samples are incubated for 5 min at 95 °C. For Western blotting, proteins are separated by SDS-PAGE, blotted and challenged with anti-Squid antibody and then with anti-Hrb87F antibody. Primary antibody binding is detected using SuperSignal West Femto substrate (Pierce). Chemiluminescent signals are acquired with the ChemiDoc XRS imager (BioRad).

## 4. Conclusions

We have improved the previously developed method by La Rocca to prepare nuclear and cytoplasmic protein extracts starting from small quantities of WL and/or several *Drosophila*’s tissues in a single extraction (Figure 1A). The first part of La Rocca protocol is based on purification of cells and nuclei from a crude homogenate and of cytoplasmic proteins in the supernatant fraction, using several manual steps and a large number of larvae (mg of larvae). In order to wash off the excess of food debris from larval bodies much time is spent and nylon membranes are needed to use. Our protocol is very fast and do not require a large number of larvae as well as washing and filter steps. The second part of La Rocca protocol is based on purification of a supernatant containing the larval nuclear extract, using dounce and at least 45 min of incubation time. We propose a protocol faster in which any dedicated instrument is not required and then we settled an efficient buffer (NEB) to fast allow nuclear homogenate and easily recover proteins from nuclear fraction. The main change that makes our protocol very useful is the introduction of WASH150 and WASH250 buffers and of two fast centrifugation steps. The rationale of the introduction of a gradient of sucrose until 250 mM using WASH150 and WASH250 buffers, ensure a pull down of intact nuclei and centrifugation steps allow a complete removal of proteins of cytoplasmic fraction as well as cytoplasmic organelles.

The nuclear and cytoplasmic extracts produced with our protocol are highly stable as shown by Western Blot and by co-immunoprecipitation assay. Moreover, the yield of our protocol is really good as starting from 10 g of WL, using La Rocca protocol is possible to produce of larval nuclear protein extracts with an average yield of 2.5 mg/mL [7]. Instead using our protocol, starting from really small quantity of *Drosophila*’s whole larvae it is possible to produce in a single extraction 200 μg of cytoplasmic protein extracts and 20 μg of nuclear protein extract (Figure 1B1). At the same time, starting from tissues (considering average between MT, SG and B), it is possible to produce almost 37 μg of cytoplasmic protein extracts and 3.65 μg of nuclear protein extracts (Figura 1B2). We are confident that this protocol will be really useful to the fly community, not only since in a single extraction it is possible to obtain nuclear and cytoplasmic proteins from whole larvae and larval tissues sources, but also because this method could be fundamental for those proteins that are able to shuttle between the nuclear and cytoplasmic compartments at the same time. In this way, it could be possible to complement *in vivo* protein function studies and biochemical analyses.
